# Contact Toxicity and Residual Efficacy of Indoxacarb against the European Earwig (Dermaptera: Forficulidae)

**DOI:** 10.3390/insects3030593

**Published:** 2012-06-25

**Authors:** Susan C. Jones, Joshua L. Bryant

**Affiliations:** Department of Entomology, Ohio State University, 2501 Carmack Rd., Columbus, OH 43210-1065, USA; E-Mail: bryant.1310@osu.edu

**Keywords:** *Forficula auricularia*, oxadiazine, pesticide, residual insecticide, urban environments

## Abstract

Indoxacarb (Arilon 20WG) was evaluated against a nuisance pest, the European earwig (*Forficula auricularia*), and was found to be an effective contact toxicant with residual activity on substrates commonly encountered in urban environments. Within 16 h of being directly sprayed with indoxacarb, ≥90% of earwigs from two populations were either ataxic, moribund, or dead, and 100% displayed these symptoms of severe intoxication at 1 d. Brief exposure (5 min or 1 h) to dried residues on either a porous (pine wood) or non-porous (ceramic tile) substrate also was sufficient to cause severe intoxication of earwigs within 1 d. In all bioassays, indoxacarb-treated earwigs showed no signs of recovery during the 21-d observation period. In outdoor urban habitats, intoxicated earwigs would be more vulnerable to desiccation, predation, or pathogens leading to higher mortality than in a laboratory setting.

## 1. Introduction

The European earwig, *Forficula auricularia* L. (Dermaptera: Forficulidae), was initially reported from North America in the early 1900s, and extensive outbreaks were first noted in Newport, RI, in 1911 and in Seattle, WA, in 1915 [1]. Populations subsequently exploded near harbors suggesting an introduction by ships, presumably from infested nursery stock from Europe [2]. 

The European earwig apparently has been introduced multiple times into North America [3,4]. Molecular and behavioral evidence suggests that European populations and North American introductions may consist of two as of yet unrecognized sibling species (species A and B) in the *F. auricularia* complex [3,4]. Both sibling species produce a single clutch of eggs during the winter, but species B produces another additional clutch in the spring after dispersal of the first nymphal cohort [3]. 

European earwigs have one generation per year. Earwigs overwinter as adults, although adult males seldom survive to overwinter in northern climates [1]. During the winter, the female lays eggs in an underground burrow where she protects the eggs and cares for the early instar nymphs, which leave the nest during springtime within about a month after hatching [5,6,7]. Nymphs subsequently are active in the soil and aboveground. Four nymphal instars precede the adult stage, which develops by summer. Large numbers of earwigs often congregate in shelters during the day.

European earwigs are nocturnal scavengers that primarily feed on decaying organic matter, plant tissues, and live or dead insects, but they occasionally may be predaceous or herbivorous. As omnivores, earwigs can have dual status as beneficial insects as well as pests. In the agricultural landscape, earwigs are important predators of many crop pests, including aphids, mites, and other soft-bodied arthropods [8], and hence can serve as biological control agents. Conversely, earwigs can directly damage crops by feeding on flowers, fruits, and foliage [1,9,10,11]. 

European earwigs often are abundant in the urban landscape, where they favor moist habitats such as mulch, the underside of pavers and rocks, and the exterior foundations of homes. Moisture is critical to their survival. Large earwig populations can develop during favorable conditions. Earwigs live outdoors and do not establish themselves indoors, but during dry, hot weather, many of these insects enter homes, where their presence is objectionable. They are nuisance pests that are not readily tolerated in homes, particularly in high numbers. Although practically harmless, earwigs have a fearsome appearance due to their habit of flexing the abdomen and displaying their pincer-like forceps. They can inflict a sharp pinch if carelessly handled. Furthermore, some earwig species secrete foul-smelling substances and quinones, which can cause minor skin abrasions in humans. Hence, many opt for earwig control in urban environments.

Pyrethroids are the predominant insecticide class in over-the-counter (OTC) products and professional use products for control of earwigs and other household pests. Colvin and Cranshaw [12] evaluated OTC pyrethroids and carbaryl, whose labels list many target pests. When earwigs were exposed to wet insecticide deposits on filter paper, the majority were moribund or dead after 3 h exposure to permethrin, bifenthrin, and carbaryl, whereas low mortality was evident with esfenvalerate. In a second experiment, deltamethrin was the most efficacious insecticide at the labeled rate, with earwigs experiencing 95% mortality after 48 h, whereas five other products (bifenthrin, permethrin, lambda-cyhalothrin, carbaryl, and esfenvalerate) resulted in lower mortality (75, 52, 35, 17.5, and 0%, respectively) during the same period [12].

Other studies pertaining to chemical control of the European earwig have focused on either agricultural ecosystems or the non-target effects of agricultural insecticides. In a soil contact toxicity study, second instar earwigs exposed to cypermethrin and deltamethrin had low mortality when exposed to field application rates, suggesting that these rates were too low to provide control or their high adsorption coefficients reduce their bioavailability. Additionally, adult earwigs were unaffected by cypermethrin [13]. A field study investigating the effects of insecticides on non-target organisms in kiwifruit production found that mineral oil with Bt (*Bacillus thuringiensis*) had no residual effect on earwigs, whereas diazinon residues on twigs and leaves killed up to 50% of earwigs, even 10 d after treatment [14]. A study in apple orchards indicated that indoxacarb, an oxadiazine, reduced earwig populations by 76% within 2 wk of application relative to control plots [15,16]. Additionally, a study of pesticide residues on apple leaves confirmed that indoxacarb was an effective pesticide against the European earwig [17].

Indoxacarb is a pro-insecticide that is taken up either through the cuticle or via ingestion by the insect. It then is biologically activated during metabolism. The target site of the bioactive metabolite is the voltage-gated sodium channel. Insect species vary in the speed at which they metabolize indoxacarb to the decarboxylated form, thus each species varies in speed of intoxication [18]. 

Indoxacarb was registered in 2000 by the U.S. Environmental Protection Agency (EPA) and designated as a “reduced risk” pesticide because of its relatively low toxicity and favorable environmental profile; it is considered to be a replacement for organophosphates [19]. Indoxacarb has low to moderate chronic and acute toxicity and does not cause mutagenic, carcinogenic, developmental, or reproductive effects [19]. Its toxicity profile makes it suitable for use in urban environments. 

A pest control formulation with indoxacarb as the active ingredient was approved for use by EPA and introduced in 2009 as Arilon. The label specifies that the product is for use in urban habitats for control of ants, cockroaches, and nuisance pests including crickets, pillbugs, and sowbugs. Because earwigs are not among the listed nuisance pests, the current laboratory study was conducted to evaluate indoxacarb as a contact and residual insecticide against the European earwig. Insects were briefly exposed to dried residues of indoxacarb on wood and glazed tile surfaces, both of which are representative substrates encountered in urban environments.

## 2. Experimental Section

### 2.1. Earwigs

Two field-collected populations of European earwigs were used in this study. The populations were collected 7–23 June 2011, from Columbus, OH, and from Piketon, OH, on 2 July 2011. Earwigs were placed in plastic holding containers and supplied with tropical fish flakes (Tetra Holding Inc., Blacksburg, VA) and potato halves; water was provided via a test tube plugged with cotton.

### 2.2. Insecticide

A 0.05% suspension was prepared using 9.4 g (0.33 oz) of indoxacarb (Arilon 20 WG) in 3.78 L (1 gallon) of distilled water. Indoxacarb was applied using a hand pump spray bottle, with each full pump providing an average discharge of 0.965 mL to an area of 232.6 cm^2^. This yielded a treatment rate of 4.16 L/100 m^2^ (1.02 gallons/1000 ft^2^) or 20.23 mg AI/m^2^.

### 2.3. Contact Toxicity Bioassays

Indoxacarb (treatment) or water (control) was sprayed directly onto groups of five adult earwigs from each population. Each group was contained in a petri dish whose walls had been coated with fluon (Insect-a-Slip Insect Barrier, BioQuip Products, Rancho Dominguez, CA, USA) to prevent insect escape. The sprayed earwigs were immediately transferred to an untreated, lidded petri dish containing a 9 cm filter paper disc (Whatman no. 1). Food and water were provided *ad libitum* throughout the evaluation period. For each population, four replicates were established for the treatment and the control.

### 2.4. Residual Bioassays

Long-lasting effects of indoxacarb were evaluated on both a porous substrate (pine wood) and non-porous substrate (glazed ceramic tile) (U.S. Ceramic Tile, Miami, FL). Indoxacarb was applied to 15.25-cm squares of both substrates at rates of 4.08, 8.15, and 16.30 L/100 m^2^ (1, 2, and 4 gallons per 1,000 ft^2^) and allowed to dry completely (4 h). The lowest indoxacarb rate represents that typically used indoors whereas the two higher rates typically are used outdoors. The control was treated with water at the highest application rate. For each substrate, four replicates were established for each of the three indoxacarb rates and the control. 

In residual bioassays, groups of five adult earwigs were confined within an inverted 9 cm petri dish (walls coated with fluon, as previously described) positioned on top of a substrate. Earwigs were kept in direct contact with each substrate for either 5 min or 1 h, then they were transferred to an untreated, lidded petri dish containing filter paper (9 cm dia). Food (tropical fish flakes) and water were provided to earwigs throughout the evaluation period.

### 2.5. Evaluation

For each replicate, the number of earwigs in each health category was recorded. A continuum of behavioral responses was used to categorize each earwig’s condition as defined herein:

- Healthy–when probed, the earwig moves quickly and in a coordinated manner to avoid stimulus.
- Sluggish–when probed, the earwig reacts slowly, but makes coordinated movements to avoid stimulus.
- Ataxic–when placed on its dorsum, the earwig is able to right itself, but it is unable to coordinate its movements to avoid the stimulus.
- Moribund–the earwig is incapable of locomotion and shows only slight movements of appendages or other body parts.
- Dead–when probed, the earwig shows no movement whatsoever.

At each observation time (4 h, 16 h, 1 d, 2 d, 3 d, 5 d, 7 d, 10 d, 14 d, and 21 d), earwigs in each replicate were individually assessed to determine their condition. If an earwig did not show immediate signs of movement, it was gently probed with featherweight forceps to assess its reaction. Each earwig was placed on its dorsum to determine whether it could assume normal posture (ataxic *versus* moribund).

### 2.6. Data Analyses

Earwigs that were categorized as ataxic, moribund, or dead were aggregated and referred to as ‘critically affected.’ The percentage of critically affected earwigs then was transformed using arcsine square root (angular transformation). These data were analyzed in Statistica 6.0 [20] using the repeated-measures ANOVA with population, treatment, exposure time, and substrate as independent variables. Data for each observation time were evaluated as a repeated-measures dependent variable.

## 3. Results and Discussion

### 3.1. Contact Bioassays

There were no significant differences in responses of the two earwig populations, Piketon and Columbus (*F* = 1.04, df = 1, 12, p = 0.33), so data were combined for analyses. Within 16 h of being directly sprayed with indoxacarb, 93% of earwigs were ataxic, moribund, or dead, and 100% of the earwigs displayed similar symptoms of severe intoxication at the 1-d observation. Although onset of symptoms was rapid, progression to death was relatively slow, and dead earwigs did not predominate until the 21-d observation. Recovery of directly sprayed earwigs was not evident during the 21-d observation period. In contrast, in soil contact bioassays with cypermethrin and deltamethrin, some earwigs recovered after initially experiencing knock down [13]. 

### 3.2. Residual Bioassays

Substrate was not a significant factor as earwigs equally succumbed to indoxacarb residues on a porous and non-porous substrate (*F* = 1.25, df = 1, 96, p = 0.27). Additionally, population did not significantly influence the percentage of earwigs affected by treatment (*F* = 1.01, df = 1, 96, p = 0.32). Therefore, substrate and population data were pooled for analyses. 

Treatment and exposure time were significant factors (*F* = 2768.7, df = 3, 120, p < 0.001 and *F* = 27.16, df = 1, 120, p < 0.001, respectively) in terms of the percentage critically affected earwigs, with the initial onset of symptoms typically slightly slower when earwigs were exposed to indoxacarb for 5 min compared to 1 h, but all earwigs were critically affected after 1 d (Table 1). In contrast, just a few control earwigs were ataxic, moribund, or dead during the 21-d bioassay period. The treatment x observation interaction was significant (*F* = 95.0, df = 27, 1080, p < 0.001) because more adverse effects were observed at 16 h and 1 d for the highest indoxacarb rate (16.30 L/100 m^2^) compared to the lower rates (4.08 and 8.15 L/100 m^2^). The treatment x exposure time x observation interaction was significant (*F* = 4.11, df = 27, 1080, p <0.001) because the percentage of critically affected earwigs at the 16-h and 1-d observations overlapped depending on indoxacarb exposure time and rate. At both the 16-h and 1-d observations, comparable data were obtained for longer exposure (1 h) to the lowest application rates (4.08 and 8.15 L/100 m^2^) and shorter exposure (5 min) to the highest rate, 16.30 L/100 m^2^ (Table 1). At 2 d and thereafter, all earwigs were critically affected regardless of indoxacarb application rate and exposure time (Table 1). Furthermore, earwigs that were briefly exposed to dried indoxacarb residues showed no signs of recovery during the 21-d observation period. In contrast, in controls, the vast majority of earwigs were healthy at all observations. 

In contact bioassays as well as in residual bioassays, the progression from ataxia or moribundity to death was relatively slow for earwigs, commencing around day 7, and >50% mortality was not observed until 21-d post-treatment. Hence, many intoxicated earwigs survived for weeks despite being critically affected by indoxacarb. However, in outdoor habitats, it is expected that intoxicated earwigs would be quite vulnerable to desiccation, predation, or pathogens, which would lead to much higher mortality (albeit secondary) than was observed in a laboratory setting. This expectation is supported by previous field research with indoxacarb [21] wherein ataxic European earwigs were observed on and/or underneath several carpenter ant-infested landscaping trees that had been sprayed with 0.1% indoxacarb (1.5 m up the trunk and 1.5 m around base). Although ataxic earwigs were observed 1-d post-treatment, none was subsequently found during a 28-d observation period. These field observations and results from the current study suggest that indoxacarb could provide control of earwigs in and around structures.

Other studies with indoxacarb likewise have shown that this insecticide is a slow-acting toxicant against various urban insect pests. Intoxication of termites [*Reticulitermes flavipes * (Kollar)] followed a predictable sequence of disorientation, ataxia, and moribundity followed by death over a period of 8 to 23 d depending on concentration and exposure time [22]. In the German cockroach [*Blattella germanica* (L.)], untreated cockroaches succumbed after being exposed to bait-fed cockroaches; mortality was attributed to consumption of the excretions and regurgitate of indoxacarb-intoxicated cockroaches as well as cannibalism. Furthermore, the horizontal transfer of indoxacarb continued beyond secondary mortality, with significant tertiary mortality observed [23].

**Table 1:** Mean percent critically affected (ataxic, moribund, or dead) earwigs at various times after brief exposure (5 min or 1 h) to dried residues of 0.05% indoxacarb on pine wood and ceramic tile (N = 16 replicates per cell).

Exposure Time	Application Rate		Critically Affected Earwigs (%)*			
	(L/100 m^2^)	(mg AI/m^2^)	4 h	16 h	1 d	2 d
5 min	4.08	20.23	0a	66b	98cdef	100h
	8.15	40.46	1a	68b	99cde	100h
	16.30	80.92	1a	94c	100gh	100h
	control	0	0a	0a	0a	0a
1 hour	4.08	20.23	1a	98cd	100efgh	100h
	8.15	40.46	6a	95cd	100fgh	100h
	16.30	80.92	6a	100defg	100h	100h
	control	0	0a	0a	0a	0a

* Means followed by a different letter are significantly different (Tukey HSD, α = 0.05).

## 4. Conclusions

Indoxacarb was an effective insecticide against the European earwig, both as a direct spray and as a residual deposit on porous and non-porous substrates commonly encountered in urban environments. All earwigs were ataxic, moribund, or dead within 1 d after being directly sprayed with indoxacarb, and the majority were similarly affected after briefly contacting (5 min or 1 h) dried indoxacarb residues on pine wood and ceramic tile. Recovery of directly sprayed earwigs or those exposed to dried residues was not evident during the 21-d observation period. Hence, indoxacarb, an oxadizine, has good potential for earwig control in urban environments.
